# Tobacco smoke particles and indoor air quality (ToPIQ-II) – a modified study protocol and first results

**DOI:** 10.1186/s12995-015-0047-8

**Published:** 2015-02-15

**Authors:** Alexander Gerber, Alexander V Hofen-Hohloch, Johannes Schulze, David A Groneberg

**Affiliations:** Institute of Occupational, Social and Environmental Medicine, Goethe-University, Theodor-Stern-Kai 7, Haus 9b, 60590 Frankfurt am Main, Germany

**Keywords:** Particulate matter, Cigarettes, Automatic environmental tobacco smoke emitter

## Abstract

**Background:**

Environmental tobacco smoke (ETS)-associated particulate matter (PM) has to be seen as an independent health hazard and needs to be discussed separately from the already well-known toxic and carcinogenic compounds contained in cigarette smoke. We believe that brand-specific amounts of PM are of public interest and should be investigated.

**Methods:**

An automatic environmental tobacco smoke emitter was developed and placed into a glass-chamber to generate cigarette smoke as reliably as possible. Cigarettes were smoked automatically according to a standardized protocol. Mean concentrations (C_mean_) and area under the curve (AUC) of PM2.5 released by the brands P&S, Virginia (without filter) and the 3R4F standard research cigarette of the University of Kentucky, USA, were measured and compared with each other.

**Results:**

C_mean_ PM2.5 of 3R4F reference was 1,725 μg/m^3^, for P&S: 1,982 μg/m^3^ and for Virginia without filter: 1,525 μg/m^3^. AUC PM2.5 for 3R4F reference was: 527,644 μg/m^3^×sec, for P&S: 606,171 μg/m^3^×sec, and for Virginia without filter: 464,788 μg/m^3^×sec.

**Conclusions:**

Our modified ToPIQ-II study protocol shows significant brand-specific differences in the amounts of PM2.5 released by cigarettes into the environment, when compared to 3R4F reference cigarettes. We believe that information about PM-release of all relevant brands in relation to reference cigarettes should be published. In the light of PM as an independent risk factor for morbidity and mortality, this may serve as a basis for further epidemiologic investigations.

## Introduction

Health effects of air pollution have been the subject of scientific research for decades, and airborne particulate matter (PM) is one important aspect of air pollution and generally considered a great health hazard [1].

PM is the term for a mixture of microscopic solid and liquid matter suspended in the air. It is attributable to both natural sources — such as volcanoes, dust storms or fires —, and to human activities (anthropogenic PM), such as the burning or fossil fuels, traffic, industrial processes or tobacco smoke. Composition, size and shape of PM depend on its source. PM can be made up of hundreds of different chemicals. The United States Environmental Protection Agency (EPA) has defined, categorized and regulated inhalable PM (particles up to 10 micrometers) since 1987 in the National Air Quality-Standard for Particulate Matter (PM-Standard) [2]. Two groups of inhalable particles are distinguished: “Inhalable coarse particles” with aerodynamic diameters larger than 2,5 μm but smaller than 10 μm, and “fine particles” with aerodynamic diameters of 2.5 μm and less. All inhalable particles smaller than 10 μm are categorized as PM10, while inhalable particles smaller than 2.5 μm are categorized as PM2.5. Their potential to cause harm depends on size, shape and solubility. The smaller the particles are, the deeper they can be inhaled. Coarse particles generally pass nose and throat and enter into the lungs. Fine particles tend to penetrate into the gas exchange regions of the lung and some may even enter into the bloodstream [3]. Surface area and solubility limit the extent of absorption of gasses and vapors. Depending on their origin, fine particles may serve as carriers for carcinogens like benzopyrenes, by absorbing them on their surface. Both long-term and short-term exposure to inhalable PM are associated with increased cardiovascular-, pulmonary- and neurological morbidity and mortality as well as with premature delivery, birth defects and premature death [4-10].

According to the “Global Burden of Disease study”, exposure to air pollution and particulate matter ranks as one of the top ten risk factors for health globally [11]. WHO recommended critical values for PM concentration in public environments are exceeded regularly [12]. While indoor particulate matter air pollution by solid fuel use for cooking is a major problem in developing countries and according to the WHO, 4.3 million people a year die from the exposure to household air pollution [13], second-hand smoke exposure is a leading contributor to the disease burden in high-income countries [14]. Apart from many toxic substances contained in ETS, epidemiological data suggests particulate matter itself to be a harmful component of ETS and demonstrates its association with many detrimental effects on human health, such as increased morbidity and mortality [5-7,15].

Therefore, not only information about nicotine and tar yield of a cigarette are of interest for the consumer. Also, information about the amount of PM emitted by a cigarette should be published, as PM seems to be an independent risk factor.

Several studies have shown a high brand loyalty, predominantly among older smokers in high-income-countries [16,17]. These smokers are likely to expose their personal environment to ETS of one brand for years or even decades. We believe that the consumer and the public have the right to be informed about brand-specific PM-amounts. In order to supply information about brand-specific PM release in relation to a 3R4F standard research cigarette, the ToPIQ-II study protocol was developed to automatically smoke cigarettes and to measure PM amounts as standardized, reliable and repeatable as possible.

## Aims

The aim of the ToPIQ-II study is to measure brand-specific amounts of PM- release of different tobacco products in a standardized way and reliably and repeatable as possible. In contrast to the original ToPIQ study protocol [18], an electric and programmable smoking machine (automatic environmental tobacco smoke emitter) is used this time to generate environmental tobacco smoke.

## Methods

### Tobacco products

Both analyzed brands (P&S and Players Virginia No. 6) are produced by Reemtsma Cigarettenfabriken GmbH, a 100% daughter company of the Imperial tobacco group. Tar and nicotine yield of Virginia without filter adds up to 10 mg and 0,8 mg. P&S cigarettes contain the same tar and nicotine yield (10 mg, 0,8 mg). The 3R4F reference cigarette has an average tar yield of 9.5 mg and a nicotine yield of 0.73 mg per cigarette.

### Automatic environmental tobacco smoke emitter (AETSE)

**Figure 1:**
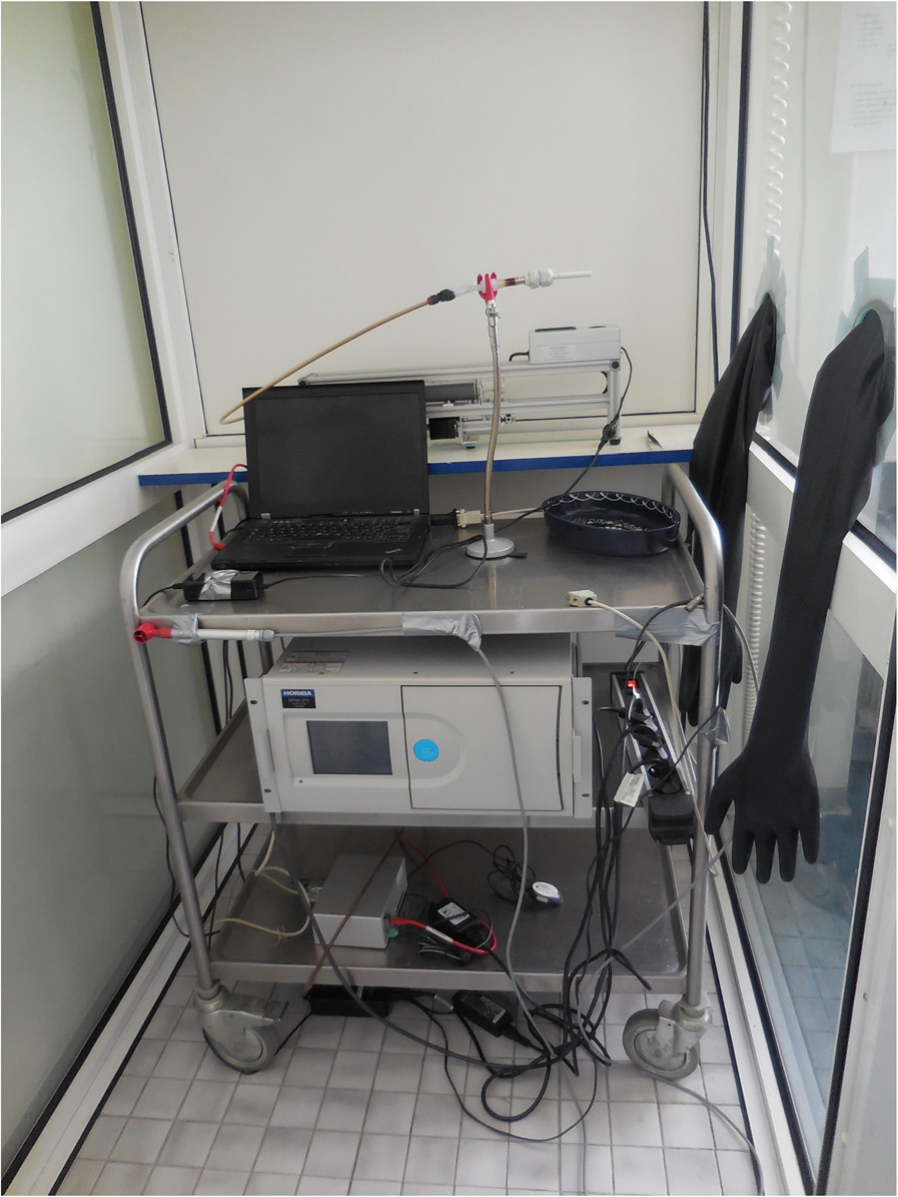
**Automatic environmental tobacco smoke emitter: the AETSE consists of a glass syringe with a plunger, a stepper motor to pull and push the plunger, a microcontroller that drives the motor and aluminium parts.** Two rubber gloves are embedded into the glass panel to operate the system.

An automatic environmental tobacco smoke emitter was developed and constructed by Schimpf-Ing, Trondheim Norway. The AETSE consists of a 200 ml glass syringe, a stepper motor, a microcontroller, aluminium profiles and mechanical parts such as hoses and valves (Figure 1). The stepper motor imitates puffing by pulling and pushing the syringe plunger, thus sucking the mainstream smoke (MS) into the syringe and exhaling it into the chamber. Igniting and extinguishing is carried out via two rubber gloves embedded into the glass wall, providing an isolated access to the chamber (Figure 1). Between puffs the smouldering cigarette continuously emits sidestream smoke (SS), which, together with the MS, forms environmental tobacco smoke (ETS). Frequency and tidal volume of the smoking machine were set according to a self-developed smoking protocol. The AETSE is placed inside a 2.88 m^3^ glass chamber in order to protect the researcher from exposure to ETS. To quantify the concentrations of particulate matter (PM2.5), an aerosol spectrometer was used (Model 11.09, Grimm Co., Ainring, Germany). The PM2.5 concentration was measured with a time resolution of 6 seconds. According to the EPA categorization from 1997, measurement of PM2.5 includes fine particles with an aerodynamic diameter of 2.5 μm or smaller. Using a special weighting function for PM measurement, particles are counted in different weightings depending on their aerodynamic diameter (particles < 0,5 μm: 100%, particles = 2.5 μm: 50%, particles > 3,5 μm: 0%). All measurements were conducted in air-conditioned laboratory rooms at temperatures of 22,7 ± 1,9°C and a humidity of 29% ± 5%. Since the chamber was placed inside our laboratory rooms, measurement procedures were not influenced by biases due to daily variations of PM environmental concentrations. Nevertheless, background PM concentrations were controlled permanently by reading the baseline before and after each measuring cycle.

### Smoking protocol

To generate environmental tobacco smoke from cigarettes in a standardized way, cigarettes were smoked according to a self-developed smoking protocol. It consists of 10 identical puffs of 3 seconds duration each. Every puff has a volume of 40 ml and is followed by 27 seconds of smouldering until the next puff is being started.

We first measured baseline values of particulate matter for 10 minutes. Then smoking started with an initial double-puff while igniting the cigarette manually with a cigarette lighter using the rubber gloves embedded into the glass wall. After 5 minutes, the cigarette was extinguished manually in a water bowl and baseline reading continued for 10 minutes after extinguishing each cigarette. Each measuring cycle finished with dust extraction by means of a high performance industrial suction, installed in the backboard and roof of the chamber.

### Data processing and analysis

**Figure 2:**
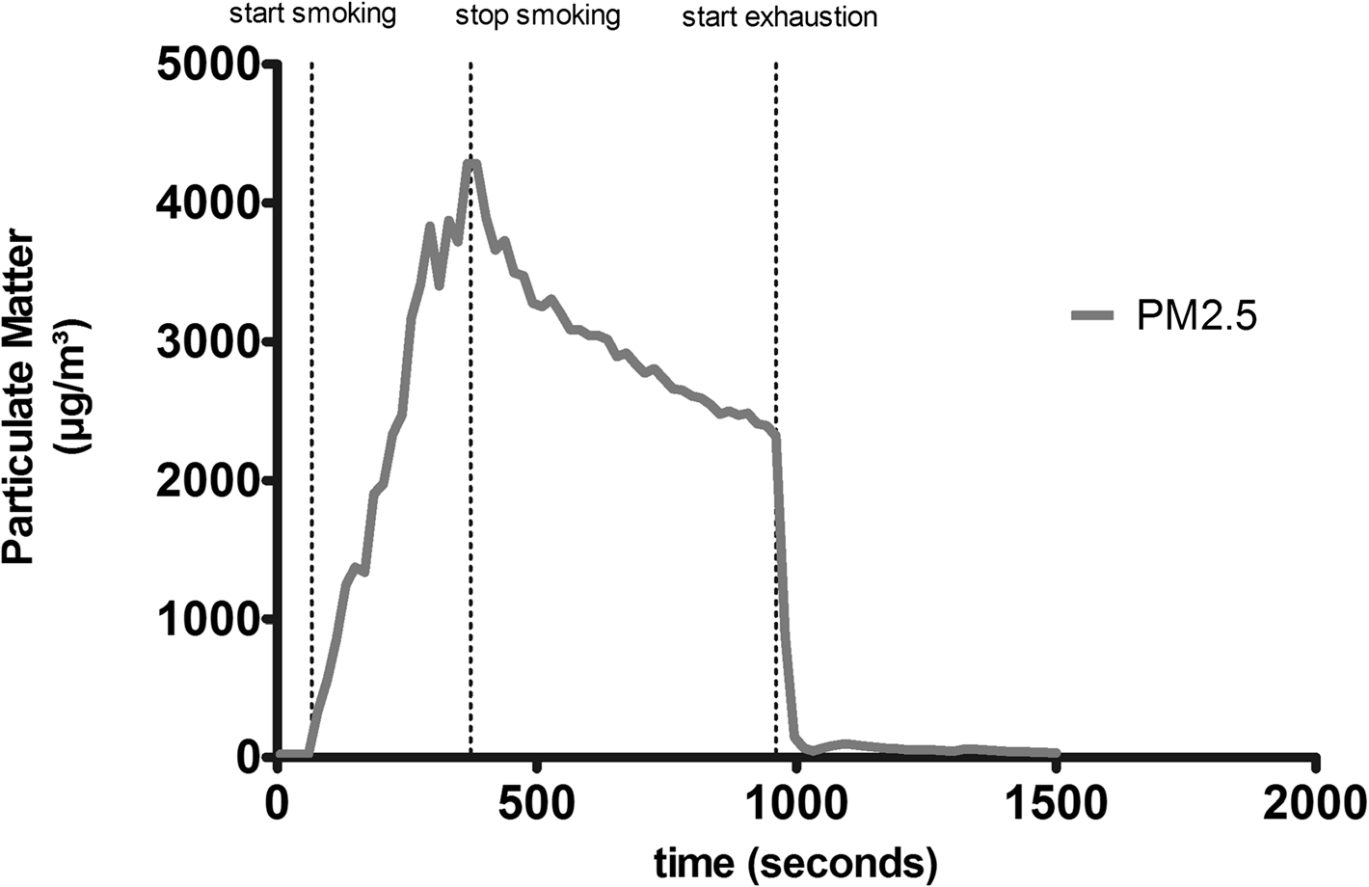
**The measuring cycle: complete measuring cycle illustrated exemplarily.**

**Figure 3:**
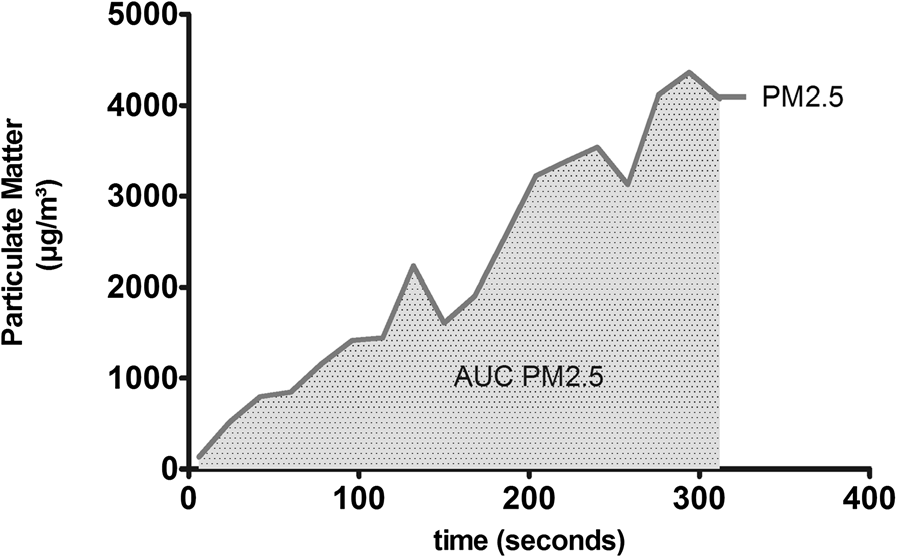
**Measurement of AUC: the interval from ignition to extinction (five minutes) was evaluated.**

**Figure 4:**
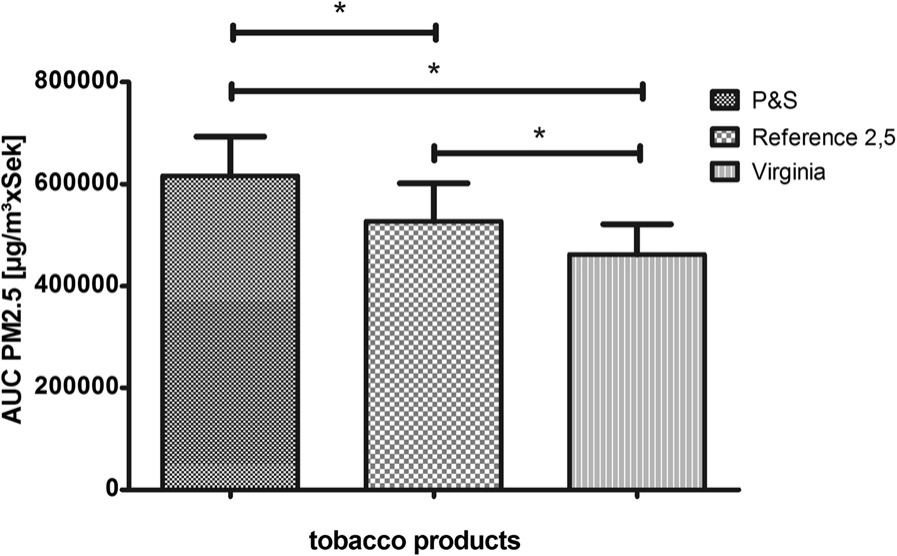
**Comparison of 3R4F, P&S and Players Virginia No. 6.** Significance was assumed, when p< 0,05 (* means significant): PM2.5 Mean concentrations are compared.

**Figure 5:**
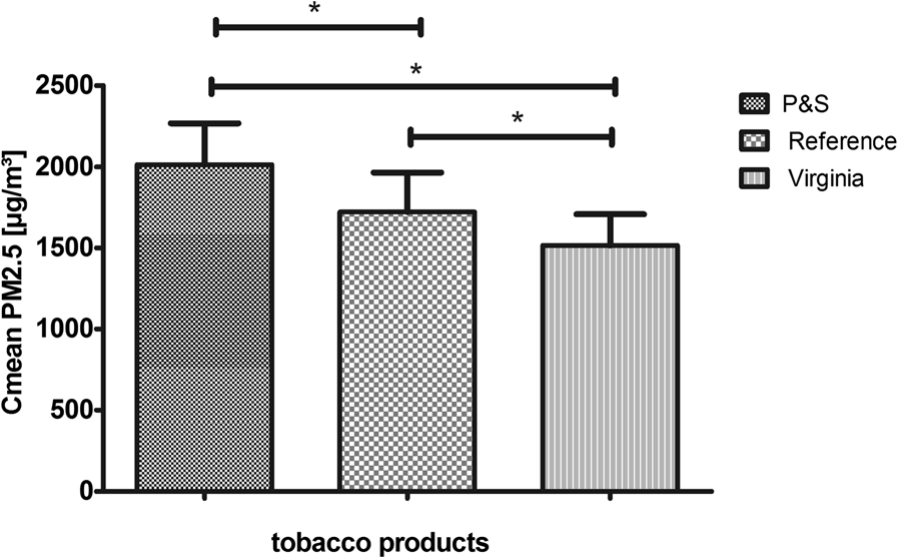
**Comparison of 3R4, P&S and Players Virginia No. 6.** Significance was assumed, when p< 0,05 (* means significant): PM2.5 AUC are compared.

The timespan between ignition and extinction of each smoking cycle (Figure 2) was analyzed and the area under the curve (AUC) of PM2.5 (AUC-PM2.5) and mean concentrations of PM2.5 (PM2.5) were calculated (Figure 3) (Graph Pad Prism 5.03). Results for the ETS exposure parameters C_mean_ (Figure 4) and AUC (Figure 5) from 40 3R4F reference cigarettes, 19 P&S cigarettes and 19 Virginia (without filter) cigarettes were tested for significant differences between the cigarette types. The one-sample t-test was used to compare each tobacco product with the reference cigarette and Bonferroni’s correction was used to counteract the problem of multiple comparisons. Significance was assumed when p < 0,05. To ensure that equal amounts of reference cigarettes and brand cigarettes being smoked for our test, we smoked two packets of 3R4F and one packet of each brand cigarette. A Gaussian distribution of individual parameters was found for all tested brands.

## Results

We found significant differences between the tested brands for C_mean_ PM2.5 and AUC PM2.5, respectively (Figures 4 and 5, Table 1).

**Table 1:** **Shows average PM2.5 mean concentrations, average AUC PM 2.5 and tar- yield for 3R4F reference cigarettes, P&S cigarettes and Virginia cigarettes without filter**

**Tobacco product**	**C**_**mean**_ **PM2.5 (μg/m** ^**3**^ **)**	**AUC PM2.5 (μg/m** ^**3**^ ** × ** **sec)**	**Combustion time (seconds)**	**Tar yield per cigarette (mg)**
3R4F reference	1,725 ± 244	527,644 ± 74,812	300	9,5
P&S	1,982 ± 252	606,171 ± 77,311	300	10
Virginia	1,525 ± 193	464,788 ± 59,417	300	10

PM concentrations of the P&S brand cigarette exceeded the reference cigarette values significantly (p < 0,05) (Figures 4 and 5). For all parameters and brands, individual values were statistically distributed according to a Gaussian distribution. Surprisingly, PM values of Virginia without filter were significantly lower than the corresponding values of 3R4F research cigarettes and P&S cigarettes (p < 0,05). This means that the mere existence of cigarette filters might have less influence on PM release than other aspects of cigarette designs, such as tobacco mixture, cellulose content and other additives that influence burning-characteristics.

## Discussion

To our knowledge, assessments of fine particulate matter between cigarette brands, as issued in our current ToPIQ-II study, have not been carried out so far. In some studies ETS-associated PM was measured. In those studies, however, PM-amounts were not compared between brands and occasionally, human smokers were made to sit in test chambers in order to produce the needed environmental tobacco smoke [19-21]. While human smokers guarantee a realistic ETS-generation, it endangers the health of the test-smokers. In our ToPIQ-II study protocol, an AETSE is used to ensure that no humans be exposed to health-hazarding ETS. Besides the unrealistic simplification of human smoking behavior, another methodological weak point of using an AETSE to generate environmental tobacco smoke is the composition of mainstream smoke exhaled by a smoking machine compared to mainstream smoke exhaled by a real smoker. In our study we assessed ETS, which consists to about 85% of sidestream smoke, released directly into the chamber by the burning cigarette and to about 15% of mainstream smoke, exhaled by the AETSE. Real smokers retain approximately 30-66% of the particulate phase contained in the MS, when puffing a cigarette, and the amount of particulate absorption by the smoker’s respiratory tract is related to size and solubility of the substance [22]. Sahu et al. compared the particle size distribution of MS and exhaled MS to predict the likelihood of deposition in the human respiratory tract. They found a ratio of the calculated deposition fraction of 0,613 for MS and 0,471 for exhaled MS [23]. The particulate phase of exhaled cigarette smoke showed a growth factor of 1.5 ± 0,3 due to coagulation and hygroscopic growth of smoke particles in the respiratory system. The AETSE most likely does not alter MS during ventilation to a comparable degree. One may ask why we did not use an internationally accepted smoking regime (e.g. the ISO machine smoking regime or the Canadian Intense). As mentioned above, our experiments were not intended to provide absolute PM data for defined situations, but rather to enable a comparison of different brands and tobacco products. We cannot compare our findings in a 2.88 m^3^ chamber with the findings of other groups, and our own health ethics don’t allow us to imitate real-life smoking conditions. Therefore, and considering that there is no really approved standardized and non-controversial method — as above mentioned protocols have been heavily criticized [24]—, we find it legitimate to use a self-developed protocol to smoke cigarettes in a standardized way according to our requirements. Despite the large brand-specific differences in ETS formation, heterogeneity between individual packs of the same brand was low. Normally, a power analysis is performed in advance to determine sample size needed to prove significance. Contrary to this usual procedure, we decided not to do so, since this being a pilot study and we could not predict the differences between the groups to be expected. However, the null hypothesis can be rejected and thus, the sample size must have been sufficient. A Gaussian distribution of individual results and a standard deviation of <15% for all brands supports our believe that our protocol, when used for experimental purposes as in our setup, is practicable and liable. A potential disadvantage of our approach is that our presently used AETSE, performing a standardized smoking protocol, is not able to adapt automatically to a possible heterogeneity of flow resistance between the different tobacco products, as human smokers would do by increasing suction power, puff volume of puff frequency to achieve the usual dosage. As cigarettes are filled with machine-cut tobacco and cellulose as binder and stabilizing components, flow resistance can vary considerably between brands and even between cigarettes of the same brand, as we found out. Using the AETSE, a large flow resistance would lead to smaller C_mean_ PM and a longer smoke duration. Comparing fix 300 seconds time intervals may lead to differently-advanced smoked cigarettes, and C_mean_ and AUC could not be compared seriously. Under real-life conditions, smokers usually smoke their cigarettes or cigarillos until they are finished. They do not adhere to an exact time interval. As P&S and Players Virginia No. 6 cigarettes show similar flow resistances and were in average equally-advanced burned down after 300 sec., we considered a fixed time interval reasonable for our present study. For future cases with great differences in flow resistance, however, as it is occasionally seen when comparing cigarettes and low-priced cigarillos, we plan to compare fixed lengths of the tobacco products [25].

Currently, reproducible data of brand-specific PM release of tobacco products under real-life conditions are not available to consumers. We do consider them to be of general public interest.

As mentioned in the introduction, exposure to particulate matter has to be seen as an independent risk factor for morbidity and mortality. Several studies have linked the rate of hospital admissions due to cardiovascular or pulmonary reasons to the levels of short-term high impact exposure to PM [26-28]. It has been shown that long-term exposure to PM correlates with cardiovascular and respiratory morbidity and mortality [29-31]. Pope et al. have assessed the relationship between long-term exposure to fine particulate air pollution and all-cause, lung cancer, and cardiopulmonary mortality. They demonstrated statistically that PM2.5 pollution was associated with all-cause, lung cancer, and cardiopulmonary mortality and that an increase of about 10 μg/m^3^ PM2.5 was associated with approximately 4%, 6%, and 8% increased risk of all-cause, cardiopulmonary, and lung cancer mortality, respectively [32].

In the light of this, it appears to be even more necessary to assess PM2.5 data in relation to reference cigarettes for all known brands as a basis for later examinations and comparisons.

Despite several limitations in this study due to the chosen experimental method, it is still reasonable to draw insightful conclusions regarding brand-specific amounts of ETS-associated PM, and further investigations of all relevant brands should be performed.
